# Trends in Transcatheter Edge-to-Edge Mitral Valve Repair Over a Decade: Data From the MiTra ULM Registry

**DOI:** 10.3389/fcvm.2022.850356

**Published:** 2022-03-08

**Authors:** Nicoleta Nita, Leonhard Schneider, Tilman Dahme, Sinisa Markovic, Mirjam Keßler, Wolfang Rottbauer, Marijana Tadic

**Affiliations:** Klinik für Innere Medizin II, Universitätsklinikum Ulm, Ulm, Germany

**Keywords:** mitral regurgitation, transcatheter edge-to-edge mitral valve repair, epidemiology, trends, outcome

## Abstract

**Objective:**

This study sought to determine the potential change in trends in the baseline characteristics of patients with symptomatic severe mitral regurgitation who underwent transcatheter edge-to-edge mitral valve repair (M-TEER) over the last decade in a high-volume center.

**Methodology:**

The investigation included 942 symptomatic patients with moderate-to-severe and severe mitral regurgitation who underwent transcatheter edge-to-edge repair (TEER) at our institution between January 2010 and March 2021. Patients were divided into quintiles and compared separately.

**Results:**

Patients treated in the last quintile had significantly lower surgical risk (Euro Score 7.2 ± 6.8% in the last quintile vs. 10.9 ± 9.4% in the first quintile, *p* < 0.001), better New York Heart Association (NYHA) status (NYHA IV 14% in the last quintile vs. 40% in the first quintile, *p* < 0.001), lower NT-pro-BNP, and smaller left ventricle diameter than patients who were treated in the first quintile. There was no difference in age between quintiles. However, an invasive hemodynamic assessment did not show significant changes over the last decade (sPAP 51.35 ± 16.2 mmHg in the first quintile vs. 51.02 ± 14.5 mmHg in the last quintile, *p* = 0.90, pulmonary capillary wedge V wave 30.7 ± 14.8 mmHg in the first quintile vs. 27.4 ± 10.3 mmHg in the last quintile, *p* = 0.40). There is a significant trend of a gradually increasing proportion of patients with degenerative mitral regurgitation (MR) over the last 10 years (*p* < 0.001). The experience gained in the M-TEER procedure brought a significant reduction in fluoroscopy time and hospitalization duration. Medical therapy significantly changed over the last decade in terms of higher use of angiotensin receptor blockers (ARBs), lower use of angiotensin-converting enzyme inhibitors (ACEIs), and the introduction of angiotensin receptor-neprilysin inhibitors (ARNIs).

**Conclusion:**

Patients undergoing the M-TEER procedure nowadays have lower surgical risk and are treated before they develop a significant left ventricular (LV) remodeling than before. The increasing expertise on the procedure over the last decade led to a rising number of patients with complex degenerative pathology being treated.

## Introduction

Mitral regurgitation (MR) together with aortic stenosis represents the most frequent valvular heart disease in Europe and America (1, 2). Surgical repair and replacement were the only choices of treatment for these valvular diseases for many years. However, in the last 15 years, transcatheter interventions that provide aortic valve implantation and mitral valve (MV) repair became a very attractive alternative to surgery in many patients and, particularly, in those with high operative risk. The first large trial that was the most important for approval of the first transcatheter mitral edge-to-edge repair (M-TEER) system (MitraClip, Abbott Vascular) was the EVEREST trial which included both functional and primary MR (3). However, M-TEER adoption was significantly slower than transcatheter aortic valve replacement (TAVR) due to the many controversies that followed the two largest trials in this field, namely, cardiovascular outcomes assessment of the MitraClip percutaneous therapy for heart failure patients with functional mitral regurgitation (COAPT) and multicentre study of percutaneous mitral valve repair MitraClip device in patients with severe secondary mitral regurgitation (MITRA-FR), which were published with different results regarding patients with functional MR (4, 5). This resulted with the approval of different criteria for M-TEER on the opposite sides of the Atlantic. The US Food and Drug Administration (FDA) initially approved M-TEER only for patients with primary MR, whereas the European Medicines Agency (EMA) was less restrictive and approved the procedure for both functional and primary MR. In 2019, the FDA approved the MitraClip for functional MR for the first time.

The other significant limitations for wider adoption of M-TEER is the lack of long follow-up data, that this intervention only mimics the Alfieri stitch procedure, and the unknown effects of durability. TAVR provides implantation of a new aortic valve, which is not the case with M-TEER. Therefore, TAVR remains the only alternative in patients with unacceptable-high operative risk to date.

The latest European guidelines recognized the importance of M-TEER in high-risk symptomatic patients who are not eligible for surgery and who do not fulfill the criteria for the procedure, suggesting an increased chance of responding to M-TEER in both groups (functional and primary) of MR patients (1). However, the importance of M-TEER is particularly emphasized in symptomatic patients with severe secondary MR with class I of evidence (1).

Data coming from registries are very important because they represent real-world evidence. However, they are also associated with many limitations, such as differences in inclusion criteria, various management of comorbidities, missing data, and different level of skills of operators. Therefore, the single-center data that provides uniform inclusion criteria, medical management, and uniform operators to a large number of patients for a long time may be of great importance in understanding trends in M-TEER over the last decade.

The aim of the present study was to determine the demographic and clinical characteristics of a large group of symptomatic patients who underwent M-TEER in our institution between 2010 and 2021. To determine the possible trend of changes, all patients were divided into quintiles and compared separately.

## Methodology

This retrospective study involved 942 patients with symptomatic moderate-to-severe and severe MR who underwent M-TEER from January 2010 to March 2021 in our institution. The interdisciplinary heart team made decisions for the M-TEER procedure in accordance with the guidelines on valvular heart disease (2). Transesophageal and transthoracic echocardiography and invasive hemodynamic measurements were performed prior to M-TEER.

Risk factors for surgical repair of the MV were prospectively evaluated using the European System for Cardiac Operative Risk Evaluation (6). M-TEER was performed in a hybrid catheterization laboratory under general anesthesia. MitraClip (Abbott) and PASCAL (Edwards) were used for MV repair, and they were implanted under fluoroscopic and echocardiographic guidance.

Clinical and laboratory data, along with data about comorbidities and medical therapy, were taken from the medical record of each patient. International classification of diseases (ICD) 9 and 10 classifications of disease were used to determine existing medical conditions. All participants are part of the prospective MiTra ULM registry. Informed written consent was taken from each participant. The local ethical committee approved the research protocol.

Short-term outcomes were estimated during hospitalization and during the first 30 days after the intervention. These outcomes included: intra-hospital mortality, 30-day rehospitalization, 30-day mortality, and 30-day major cardiovascular events (MACE—myocardial infarction or cardiac revascularization, stroke, and cardiovascular death).

### Statistical Analysis

Continuous variables were presented as mean ± SD and were compared by the Student's *t*-test for variables that showed normal distribution. The Kruskal–Wallis test was used for the comparison of continuous variables that did not show normal distribution. Differences in proportions were compared by the χ^2^ test or Fischer's exact test as appropriate. All patients were divided into quintiles to determine possible differences in the demographic and clinical characteristics and short-term outcomes of patients over the last 10 years. Continuous variables in quintiles were compared by the ANOVA, as they showed normal distribution using the Kolmogorov–Smirnov test. Troponin T and N-terminal prohormone of brain natriuretic peptide (NT-pro-BNP) did not initially show normal distribution. Therefore, a logarithmic transformation was performed. Tukey's Honestly Significant Difference (HSD) *post-hoc* analysis was used for the comparison between different quintiles. The p-value <0.05 was considered statistically significant.

## Results

Table 1 summarizes the demographic and clinical characteristics of all the study population. Our findings showed that more females than males underwent this interventional procedure over the last 10 years (56 vs. 44%). Functional MR was more prevalent than primary MR among treated patients (61 vs. 39%). Regarding the severity of heart failure (HF) symptoms, the New York Heart Association (NYHA) class III was more prevalent than class IV.

**Table 1:** Demographic characteristics and clinical parameters of study population.

	**All patients (*n* = 942)**
Age (years)	78 ± 9
Male (%)	395 (42)
Functional MR	563 (60)
BMI (kg/m^2^)	25.7 ± 4.4
Systolic blood pressure (mmHg)	126 ± 21
Diastolic blood pressure (mmHg)	73 ± 11
NYHA class	
III (%)	696 (74)
IV (%)	246 (26)
Interventions and surgeries	
PCI (%)	433 (46)
CABG (%)	153 (16)
Mitral valve surgery (%)	17 (2)
TAVR (%)	61 (6)
Aortic valve surgery (%)	51 (5)
Comorbidities	
CAD (%)	610 (65)
Previous MI (%)	218 (23)
Hypertension (%)	741 (79)
Dyslipidemia (%)	543 (58)
Diabetes (%)	261 (29)
Atrial fibrillation (%)	591 (65)
Peripheral artery disease (%)	81 (9)
COPD (%)	108 (12)
OSAS (%)	54 (6)
Peptic ulcer disease (%)	20 (2)
Renal failure (%)	452 (48)
Acute renal failure (%)	43 (5)
Hepatic cirrhosis (%)	12 (1)
Previous cancer (%)	156 (17)
Antiarrhythmia devices	
CRT (%)	83 (8)
ICD (%)	125 (13)
Pacemaker (%)	86 (9)
Scores	
Euro score II	7.5 ± 7.3
Therapy	
ACEI (%)	415 (44)
ARB (%)	258 (27)
ARNI (%)	72 (8)
Beta-blockers (%)	810 (86)
Aldosterone antagonists (%)	432 (46)
Statins (%)	628 (67)
Laboratory	
Creatinine (μmol/l)	129 ± 72
GFR (ml/min/1.73 m^2^)	49 ± 20
NT-pro-BNP (pg/ml)	5,191 ± 6,381
Echocardiography	
LVEF (%)	49 ± 18
LVEDD (mm)	58 ± 11
LVESD (mm)	41 ± 13
Interventricular septum thickness (mm)	10.8 ± 2.4
LA (mm)	55 ± 9

*ACEI, angiotensin converting enzyme inhibitor; ARB, angiotensin II receptor blocker; ARNI, angiotensin receptor II blocker - neprilysin inhibitor; BMI, body mass index; CABG, coronary artery bypass grafting; COPD, chronic obstructive pulmonary disease; CRT, cardiac resynchronization therapy; GFR, glomerular filtration rate; ICD, implantable cardiac defibrillators; LA, left atrium; LV, left ventricle; LVEF, left ventricular ejection fraction; LVEDD, left ventricular end-diastolic diameter; LVESD, left ventricular end-systolic diameter; MI, myocardial infarction; OSAS, obstructive sleep-apnea syndrome; PCI, percutaneous coronary artery intervention; TAVR, transcatheter aortic valve replacement*.

Comorbidities, such as hypertension, dyslipidemia, coronary artery disease (CAD), and atrial fibrillation, were very prevalent in the whole population (Table 1). Kidney dysfunction was also present in almost 50% of patients. All medications that are traditionally used for the treatment of symptomatic patients with MR [diuretics, angiotensin-converting enzyme inhibitor (ACEI), angiotensin receptor blocker (ARB), aldosterone antagonists, and beta-blockers] were also prevalently prescribed to our patients. An echocardiographic examination revealed dilated left ventricles and atriums in all patients, along with mildly reduced left ventricular ejection fraction (LVEF) in the whole population (Table 1).

### Hemodynamic Measurements

Table 2 shows the hemodynamic parameters measured during cardiac catheterization performed before device implantation. Right ventricular systolic, diastolic, and mean pressures and corresponding pulmonary pressures were significantly increased in patients with MR. Mean pulmonary capillary wedge pressure and left atrial (LA) pressure were also significantly increased (Table 2). The average systemic and pulmonary vascular resistances were higher in patients with MR than in the global population (Table 2).

**Table 2:** Hemodynamic measurements in study population.

	**All patients (*n* = 376)**
Heart rate (beat/min)	74 ± 15
Mean RA pressure (mmHg)	11 ± 6
Mean RV pressure (mmHg)	25 ± 17
Systolic PA pressure (mmHg)	50 ± 15
Diastolic PA pressure (mmHg)	20 ± 10
Mean PA pressure (mmHg)	33 ± 13
Mean PCWP (mmHg)	23 ± 9
Mean LA pressure (mmHg)	19 ± 10
LV end-systolic pressure (mmHg)	133 ± 70
LV end-diastolic pressure (mmHg)	20 ± 8
Systolic BP (mmHg)	126 ± 29
Diastolic BP (mmHg)	67 ± 40
Mean BP (mmHg)	87 ± 20
SVR (dynes/seconds/cm^−5^)	2,000 ± 2,144
PVR (dynes/seconds/cm^−5^)	300 ± 255
Cardiac output (l/min)	3.8 ± 1.2
Cardiac index (l/min/m^2^)	2.0 ± 0.6
Oxygen saturation in aorta (%)	90.0 ± 4.3
Oxygen saturation in PA (%)	57 ± 9

*BP, blood pressure; LA, left atrial; LV, left ventricular; MR, mitral regurgitation; PA, pulmonary artery; PCWP, pulmonary capillary wedge pressure; PVR, pulmonary vascular resistance; SVR, systemic vascular resistance*.

### Trend Differences in the Period Between 2010 and 2021

**Figure 1:**
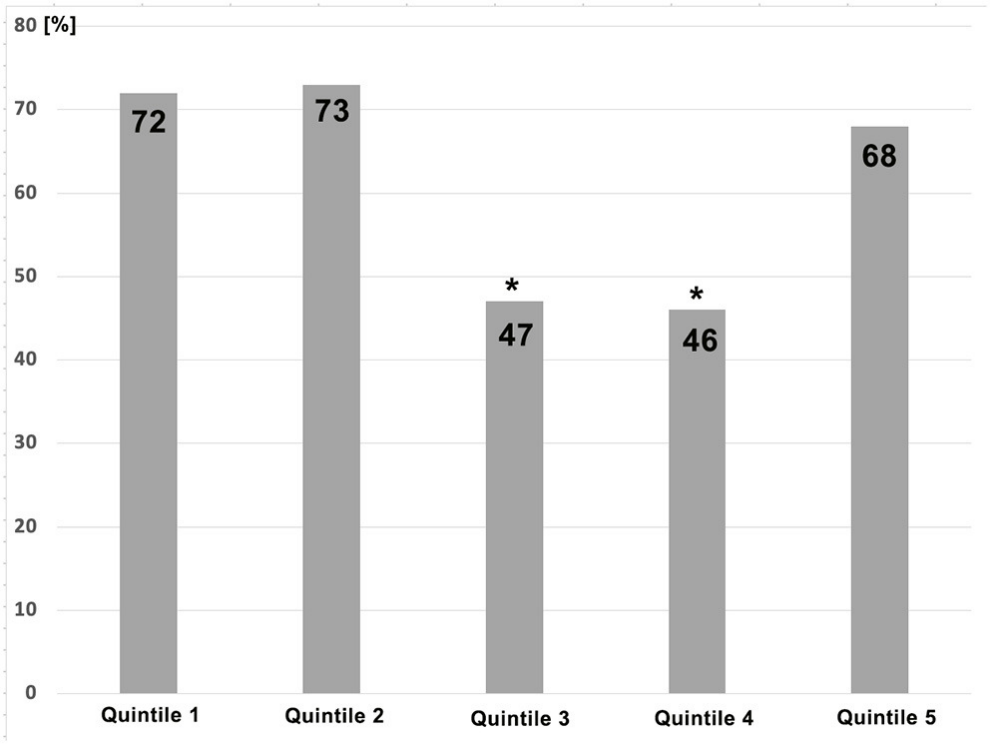
The difference of prevalence of patients with functional mitral regurgitation who underwent transcatheter edge-to-edge mitral valve repair (M-TEER) over 5 quintiles. *p < 0.05 for the difference between quintile 3 and 4 and other quintiles.

**Figure 2:**
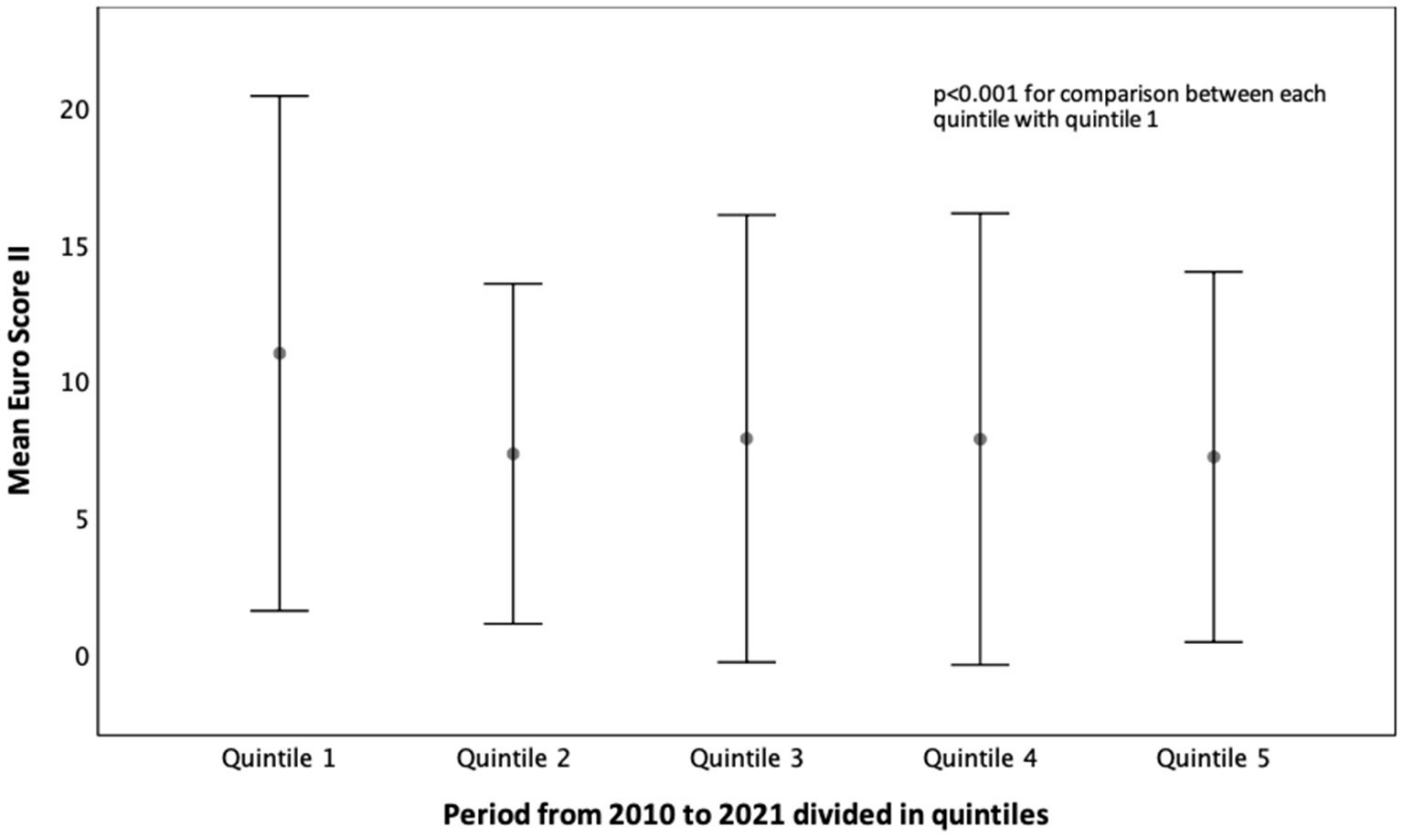
Mean Euro Score II in patients with mitral regurgitation who underwent M-TEER from 2010 to 2021.

Several interesting trends were noticed when all patients were divided into 5 equal groups (Table 3). The proportion of sexes among patients changed over the last 10 years. In the beginning, more than 60% of patients were women, whereas, in the last 2 quintiles, there was almost an equal number of women and men (Table 3). The percentage of patients with functional mitral regurgitation (FMR) gradually decreased from the first until the fourth quintile but abruptly increased in the last quintile, which interestingly corresponds with the COVID-19 pandemic (Figure 1, Table 3). The percentage of patients with NYHA class III significantly increased but that was decreased with class IV in the last two quintiles in comparison to the first experience. The same trend was noticed for CAD. Acute renal failure was significantly lower in the last quintile in comparison with the other quintiles (Table 3). The number of patients with cardiac resynchronization therapy (CRT) and ICD significantly reduced in the last two quintiles compared with initial experience. Operative risk Euro score immediately reduced after the initial experience (Figure 2).

**Table 3:** Demographic characteristics and clinical parameters in period between 2010 and 2021 separated in quintiles.

	**I (*n* = 188)**	**II (*n* = 188)**	**III (*n* = 188)**	**IV (*n* = 188)**	**V (*n* = 190)**	***p***
Age (years)	78 ± 9	76 ± 8	76 ± 9	78 ± 9	78 ± 8	0.193
Male (%)	71 (38)	75 (40)	68 (36)	92 (49)^f^	89 (47)	0.042
Functional MR (%)	135 (72)	137 (73)	88 (47)^b^	87 (46)^b^	130 (68)^d^^,^ ^g^	<0.001
BMI (kg/m^2^)	25.4 ± 4.3	26.0 ± 4.4	25.7 ± 4.4	25.9 ± 5.5	26.6 ± 5.3	0.219
Systolic blood pressure (mmHg)	121 ± 20	127 ± 19^a^	127 ± 23^a^	125 ± 19	126 ± 20	0.026
Diastolic blood pressure (mmHg)	70 ± 10	74 ± 11^b^	73 ± 14	73 ± 11	72 ± 12	0.004
NYHA class						
III (%)	113 (60)	145 (77)^b^	132 (70)	143 (76)^b^	163 (86)^b^^,^ ^d^	<0.001
IV (%)	75 (40)	43 (23)^b^	56 (30)	45 (24)^b^	27 (14)^b^^,^ ^d^	<0.001
Interventions and surgeries						
PCI (%)	81 (43)	78 (41)	89 (47)	93 (49)	92 (48)	0.486
CABG (%)	46 (24)	20 (11)^b^	35 (19)	19 (10)^b^	33 (17)	0.002
Mitral valve surgery (%)	4 (2)	1 (0.5)	5 (3)	4 (2)	3 (2)	0.620
TAVR (%)	7 (4)	16 (9)	10 (5)	14 (7)	14 (7)	0.345
Aortic valve surgery (%)	13 (7)	9 (5)	9 (5)	10 (5)	10 (5)	0.717
Comorbidities						
Previous MI (%)	40 (21)	41 (22)	58 (31)	40 (21)	45 (23)	0.283
CAD (%)	140 (74)	124 (66)	132 (70)	115 (61)^b^	113 (60)^b^	0.001
Hypertension (%)	153 (81)	156 (83)	146 (78)	148 (79)	158 83	0.558
Dyslipidemia (%)	105 (56)	117 (62)	116 (62)	101 (54)	119 (63)	0.279
Diabetes (%)	54 (29)	62 (33)	41 (22)	57 (30)	54 (28)	0.177
Atrial fibrillation (%)	126 (67)	125 (66)	122 (65)	113 (60)	120 (63)	0.534
Peripheral artery disease (%)	21 (11)	14 (7)	15 (8)	17 (9)	14 (8)	0.736
COPD (%)	23 (12)	28 (15)	22 (12)	20 (11)	17 (9)	0.518
OSAS (%)	11 (6)	12 (6)	14 (7)	8 (4)	10 (5)	0.753
Peptic ulcer disease (%)	11 (6)	3 (2)	1 (0.5)^b^	3 (2)	2 (2)^a^	0.003
Renal failure (%)	99 (53)	112 (60)	92 (49)	102 (54)	54 (28)^b^^,^ ^c^^,^ ^d^^,^ ^g^	<0.001
Acute renal failure (%)	5 (3)	10 (5)	8 (4)	16 (9)^a^	5 (2)^h^	0.041
Hepatic cirrhosis (%)	3 (2)	2 (1)	2 (1)	2 (1)	3 (2)	0.950
Previous cancer (%)	35 (19)	34 (18)	33 (18)	30 (16)	27 (14)	0.117
Antiarrhythmia devices						
CRT (%)	17 (9)	24 (13)	21 (11)	11 (6)^e^	10 (5)^e^	0.047
ICD (%)	34 (18)	34 (18)	20 (11)	19 (10)^a^^,^ ^e^	18 (9)^a^^,^ ^e^	0.016
Pacemaker (%)	18 (10)	19 (10)	15 (8)	17 (9)	17 (9)	0.966
Scores						
Euro score II	10.9 ± 9.4	7.3 ± 6.2^a^	7.9 ± 8.2^a^	7.8 ± 8.3^a^	7.2 ± 6.8^a^	<0.001
Ln (Euro score II)	2.1 ± 0.8	1.7 ± 0.7^a^	1.7 ± 0.8^a^	1.7 ± 0.8^a^	1.7 ± 0.8^a^	<0.001
Therapy						
Loop diuretics (%)	141 (75)	150 (80)	139 (74)	150 (80)	154 (81)	0.409
ACEI (%)	98 (52)	92 (49)	80 (43)	82 (44)	63 (33)^b^^,^ ^c^	0.004
ARB (%)	40 (21)	51 (27)	59 (31)	55 (29)	53 (28)	0.154
ARNI (%)	–	4 (3)	14 (7)^e^	20 (11)^b^	34 (18)^b^^,^ ^d^	0.001
Beta-blockers (%)	162 (86)	160 (85)	164 (87)	164 (87)	160 (84)	0.874
Aldosterone antagonists (%)	78 (41)	77 (41)	83 (44)	98 (52)	96 (51)	0.101
Statins (%)	107 (57)	137 (73)^b^	123 (65)	125 (66)	136 (72)^b^	0.013
Laboratory						
Creatinine (μmol/l)	128 ± 57	138 ± 77	125 ± 66	136 ± 74	123 ± 72	0.155
GFR (ml/min/1.73 m^2^)	49 ± 18	45 ± 17	52 ± 22^c^	46 ± 19^d^	51 ± 20^e^	<0.001
Troponin T (ng/l)	86 ± 38	121 ± 67	83 ± 49	41 ± 38	44 ± 41	0.626
Ln (Troponin T)	3.5 ± 1.0	3.7 ± 0.9	3.8 ± 0.7	4.2 ± 1.6	3.9 ± 0.6	0.341
NT-pro-BNP (pg/ml)	5,639 ± 6,427	4,915 ± 4,818	6,422 ± 8,566	5,302 ± 5,842	3,810 ± 4,626^d^	0.005
Ln (NT-pro-BNP)	8.1 ± 1.1	8.0 ± 1.1	8.1 ± 1.3	8.0 ± 1.1	7.7 ± 1.1^d^	0.009
Echocardiography						
LVEF (%)	44 ± 17	43 ± 18	43 ± 18	46 ± 17	43 ± 13	0.410
LVEDD (mm)	62 ± 11	60 ± 12	60 ± 11	59 ± 11	58 ± 10	0.067
LVESD (mm)	47 ± 14	46 ± 14	47 ± 14	43 ± 13^a^^,^ ^f^	42 ± 11^b^^,^ ^d^	0.029
Interventricular septum thickness (mm)	10 ± 2	11 ± 3	11 ± 2	12 ± 3	11 ± 2	0.279
LA (mm)	57 ± 10	54 ± 8	55 ± 8	55 ± 9	55 ± 11	0.230
Procedure						
Fluoroscopy time (min)	33 ± 19	30 ± 17	27 ± 17^b^	26 ± 16^b^	26 ± 15^b^	0.002
Hospitalization						
Time before procedure (days)	5.6 ± 8.0	5.1 ± 5.9	5.0 ± 4.7	3.7 ± 3.3^b^	2.9 ± 2.7^b^^,^ ^c^^,^ ^d^	<0.001
Time after procedure (days)	8.3 ± 4.8	7.0 ± 6.2	6.2 ± 6.9^b^	5.2 ± 3.0^b^^,^ ^c^	4.8 ± 2.5^b^^,^ ^c^	<0.001
ICU length (days)	1.3 ± 2.4	1.4 ± 2	1.5 ± 2.3	0.9 ± 1.5^b^^,^ ^c^	0.8 ± 1.1^b^^,^ ^c^^,^ ^d^	<0.001
Short-term outcome						
Intra-hospital mortality (%)	11 (6)	9 (5)	6 (3)	6 (3)	5 (3)	0.463
30-day re-hospitalization (%)	8 (4)	7 (4)	5 (3)	11 (6)	7 (4)	0.613
30-day MACE (%)	20 (11)	16 (9)	14 (7)	15 (8)	11 (6)	0.532
30-day mortality (%)	12 (6)	10 (5)	10 (5)	7 (4)	6 (3)	0.581

a *p* < 0.05 for comparison with I quintile.

b *p < 0.01 for comparison with I quintile*.

c *p < 0.01 for comparison with II quintile*.

d *p < 0.01 for comparison with III quintile*.

e *p < 0.05 for comparison with II quintile*.

f *p < 0.05 for comparison with III quintile*.

g *p < 0.01 for comparison with IV quintile*.

h *p < 0.05 for comparison with IV quintile*.

*ACEI, angiotensin converting enzyme inhibitor; ARB, angiotensin II receptor blocker; ARNI, angiotensin receptor II blocker - neprilysin inhibitor; BMI, body mass index; CABG, coronary artery bypass grafting; COPD, chronic obstructive pulmonary disease; CRT, cardiac resynchronization therapy; GFR, glomerular filtration rate; ICD, implantable cardiac defibrillators; LA, left atrium; LV, left ventricle; LVEF, left ventricular ejection fraction; LVEDD, left ventricular end-diastolic diameter; LVESD, left ventricular end-systolic diameter; MI, myocardial infarction; OSAS, obstructive sleep-apnea syndrome; PCI, percutaneous coronary artery intervention; TAVR, transcatheter aortic valve replacement*.

Therapy was also changed in terms of decreased percentage of ACEI and increased percentage of angiotensin receptor-neprilysin inhibitor (ARNI) usage (Table 3). The NT-pro-BNP level only reduced in the last quintile. Left ventricular (LV) remodeling did not significantly change over various quintiles, but left ventricular end-systolic diameter (LVESD) was significantly lower in the last two quintiles.

**Figure 3:**
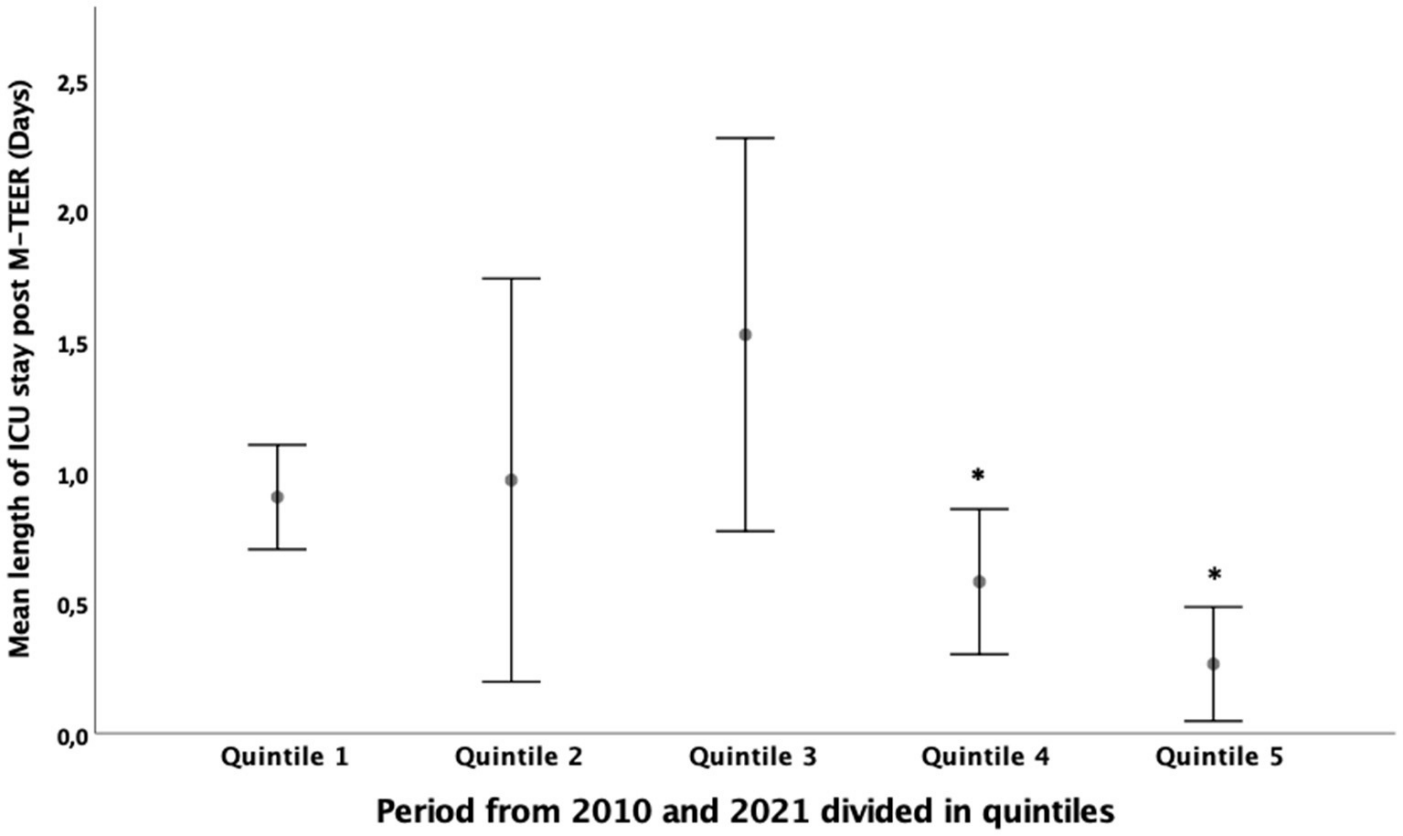
Mean length of hospitalization in the ICU of patients with mitral regurgitation who underwent M-TEER from 2010 to 2021. *p < 0.05 for the difference between quintile 4 and 5 and other quintiles.

The length of hospitalization, before and after intervention and in the ICU (Figure 3), significantly reduced in the last two quintiles (Table 3). The duration of hospitalization in ICU was directly associated with Euro Score II (β = 0.211, *p* < 0.001). Short-term outcomes (intra-hospital mortality, 30-day hospitalization, MACE, and mortality) did not change from 2010 to 2021.

### Comparison Between the First and Current Experience

To determine the differences in the characteristics and outcomes of patients between the initial experience and current clinical practice, we compared the first and fifth quintiles (Table 4). The operative risk in recently managed patients is significantly lower than it was at the beginning. The prevalence of common comorbidities was mainly similar between the two quintiles. CAD and renal failure are less prevalent in recent patients than those in the beginning. Interestingly, ACEIs are less used, and ARBs are more frequently prescribed in recently operated patients. New groups of medications (ARNI), which did not exist at the beginning, also appeared in the last quintile. Interestingly, statins are more frequently prescribed in patients in the last quintile (Table 4).

**Table 4:** Difference between the first and last quintile in demographic characteristics and clinical parameters.

	**I (*n* = 188)**	**V (*n* = 190)**	***p***
Age (years)	78 ± 9	78 ± 8	0.430
Male (%)	71 (38)	89 (47)	0.105
Functional MR (%)	135 (72)	130 (68)	0.501
BMI (kg/m^2^)	25.4 ± 4.3	26.6 ± 5.3	0.021
Systolic blood pressure (mmHg)	121 ± 20	126 ± 20	0.013
Diastolic blood pressure (mmHg)	70 ± 10	72 ± 12	0.028
NYHA class			
III (%)	113 (60)	163 (86)	<0.001
IV (%)	75 (40)	27 (14)	
Interventions and surgeries			
PCI (%)	81 (43)	92 (48)	0.392
CABG (%)	46 (24)	33 (17)	0.118
Mitral valve surgery (%)	4 (2)	3 (2)	1.00
TAVR (%)	7 (4)	14 (7)	0.163
Aortic valve surgery (%)	13 (7)	10 (5)	0.661
Comorbidities			
Previous MI (%)	40 (21)	45 (23)	0.545
CAD (%)	140 (74)	113 (60)	0.003
Hypertension (%)	153 (81)	158 83	0.781
Dyslipidemia (%)	105 (56)	119 (63)	0.234
Diabetes (%)	54 (29)	54 (28)	1.00
Atrial fibrillation (%)	126 (67)	120 (63)	0.429
Peripheral artery disease (%)	21 (11)	14 (8)	0.476
COPD (%)	23 (12)	17 (9)	0.391
OSAS (%)	11 (6)	10 (5)	1.00
Peptic ulcer disease (%)	11 (6)	2 (2)	0.023
Renal failure (%)	99 (53)	54 (28)	<0.001
Acute renal failure (%)	5 (3)	5 (2)	1.00
Hepatic cirrhosis (%)	3 (2)	3 (2)	1.00
Previous cancer (%)	35 (19)	27 (14)	0.197
Antiarrhythmia devices			
CRT (%)	17 (9)	10 (5)	0.223
ICD (%)	34 (18)	18 (9)	0.022
Pacemaker (%)	18 (10)	17 (9)	1.00
Scores			
Euro score II	10.9 ± 9.4	7.2 ± 6.8	<0.001
Ln (Euro score II)	2.1 ± 0.8	1.7 ± 0.8	<0.001
Therapy			
Loop diuretics (%)	141 (75)	154 (81)	0.198
ACEI (%)	98 (52)	63 (33)	<0.001
ARB (%)	40 (21)	53 (28)	0.029
ARNI (%)	–	34 (18)	–
Beta-blockers (%)	162 (86)	160 (84)	0.548
Aldosterone antagonists (%)	78 (41)	96 (51)	0.109
Statins (%)	107 (57)	136 (72)	0.005
Laboratory			
Creatinine (μmol/l)	128 ± 57	123 ± 72	0.496
GFR (ml/min/1.73 m^2^)	49 ± 18	51 ± 20	0.257
Troponin T (ng/l)	86 ± 38	44 ± 41	0.184
Ln (Troponin T)	3.5 ± 1.0	3.9 ± 0.6	0.123
NT-pro-BNP (pg/ml)	5,639 ± 6,427	3,810 ± 4,626	0.012
Ln (NT-pro-BNP)	8.1 ± 1.1	7.7 ± 1.1	0.009
Echocardiography			
LVEF (%)	44 ± 17	43 ± 13	0.598
LVEDD (mm)	62 ± 11	58 ± 10	0.010
LVESD (mm)	47 ± 14	42 ± 11	0.015
Interventricular septum thickness (mm)	10 ± 2	11 ± 2	0.105
LA (mm)	57 ± 10	55 ± 11	0.192
Procedure			
Fluoroscopy time (min)	33 ± 19	26 ± 15	<0.001
Hospitalization			
Time before procedure (days)	5.6 ± 8.0	2.9 ± 2.7	<0.001
Time after procedure (days)	8.3 ± 4.8	4.8 ± 2.5	<0.001
ICU length (days)	1.3 ± 2.4	0.8 ± 1.1	<0.001
Short-term outcome			
Intra-hospital mortality (%)	11 (6)	5 (3)	0.132
30-day re-hospitalization (%)	8 (4)	7 (4)	0.591
30-day MACE (%)	20 (11)	11 (6)	0.093
30-day mortality (%)	12 (6)	6 (3)	0.154

*ACEI, angiotensin converting enzyme inhibitor; ARB, angiotensin II receptor blocker; ARNI, angiotensin receptor II blocker - neprilysin inhibitor; BMI, body mass index; CABG, coronary artery bypass grafting; COPD, chronic obstructive pulmonary disease; CRT, cardiac resynchronization therapy; GFR, glomerular filtration rate; ICD, implantable cardiac defibrillators; LA, left atrium; LV, left ventricle; LVEF, left ventricular ejection fraction; LVEDD, left ventricular end-diastolic diameter; LVESD, left ventricular end-systolic diameter; MI, myocardial infarction; OSAS, obstructive sleep-apnea syndrome; PCI, percutaneous coronary artery intervention; TAVR, transcatheter aortic valve replacement*.

At present, patients undergo transcatheter MV repair at earlier NYHA classifications. Several points that confirm our conclusion are as follows: the majority of patients have NYHA class III, left ventricle and left atrium are less dilated, and NT-pro-BNP is significantly lower in recently operated patients (Table 4).

Our experience significantly influenced fluoroscopy time and hospitalization duration, which significantly decreased in the last quintile compared with the first quintile (Table 4). The reduction in hospitalization before and after M-TEER is particularly important during the COVID-19 era, which showed how the availability of hospital beds might be an important limitating factor for all non-elective interventional procedures in cardiology. Nevertheless, the short-term outcomes (intrahospital mortality, 30-day hospitalization, MACE, and mortality) did not significantly change between patients who were treated at the beginning of M-TEER and those who were treated more recently (Table 4).

## Discussion

The present study revealed several important findings that should be further discussed: (i) patients who underwent TEER had very high surgical risk and a large burden of comorbidities; (ii) hemodynamic changes in the whole population correspond with long-lasting MR; (iii) patients who underwent TEER more recently were under lower surgical risk with higher prevalence of NYHA III, lower prevalence of NYHA IV, and lower NT-pro-BNP level than patients who were treated at the beginning; (iv) the burden of comorbidities remained the same over 10 years with the exception of CAD which decreased; (v) therapy changed over the last decade in terms of higher use of ARBs, lower use of ACEIs, and introduction of ARNI in treatment paradigm; (vi) LV and LA dilatation is less pronounced in more recently treated patients than those treated initially, which also confirms the hypothesis that we use M-TEER earlier nowadays than it was before; and (vii) experience in M-TEER brought significant reduction in fluoroscopy time and hospitalization duration, but the short-term outcome remained unchanged over the last decade.

Our results regarding the baseline demographic and clinical characteristics of patients who undergo M-TEER do not significantly differ from the other institutions and investigators (7–10). According to the guidelines, M-TEER is reserved for patients with the highest operative risk. Therefore, the high prevalence of comorbidities is not surprising. The same is valid for hemodynamic changes that confirm long-standing MR in treated patients with increased LA and wedge pressures, along with elevated right-heart pressures, which correspond with previous findings (11).

The trend that we observe in everyday clinical practice regarding earlier M-TEER treatment in the last few years than at the beginning of the M-TEER experience was confirmed in the current study. Treated patients are less severe, with more prevalent NYHA III than NYHA IV, lower NT-pro-BNP and Euro Score, and less severe LV and LA dilatation than it was previously. There are several possible reasons for these findings that include more clinical experience with M-TEER, improvement of existing M-TEER device (MitraClip, Abbott), the appearance of new M-TEER device (PASCAL, Edwards) in the last couple of years, and clinical real-world data that not only confirmed the feasibility and safety of M-TEER (9) but also displayed significant improvements in short and mid-term outcomes in symptomatic patients with significant MR (12–14).

The burden of concomitant diseases did not significantly change over the last decade. The substantial reduction in the prevalence of coronary artery disease and renal failure (chronic and acute) in more recently operated patients is an important trend that was noticed in our study population. This tendency is not only related to the timely decisions about M-TEER, which is probably the reason for the lower prevalence of renal failure in recently treated patients, but also with the change in proportion between primary and functional MR. Notably, we noticed a sharp trend of reduction of functional MR in treated patients over the first four quintiles, which corresponded with an increased prevalence of primary MR. This might explain the trend of a lower percentage of coronary artery disease among patients treated with M-TEER. Interestingly, this trend was disrupted only in the last quintile, which corresponds with the COVID-19 pandemic. This might, therefore, influence the higher referral of symptomatic patients with heart failure who usually suffer from functional MR. The gradual reduction in the percentage of patients with functional MR was also associated with favorable results of studies and trials that involved patients with primary MR (4, 9, 15), along with controversial results from the European MITRA-FR trial regarding the outcome in patients with functional MR (5).

The change in the medical treatment paradigm should be particularly emphasized. Particularly, our results revealed a trend of reduction in ACEIs use and an increase in ARBs use. The introduction of new medication in the treatment of patients with heart failure, i.e., ARNI, should be acknowledged (16, 17), along with a significant trend of increase of its usage starting from the second quintile and finishing with the last quintile. Given that ARNI is only available in combination with valsartan and that many patients receive valsartan as monotherapy before initiation of ARNI, it is reasonable to hypothesize that the introduction of ARNI was an important factor for the higher prescription of ARBs in comparison with ACEIs in patients undergoing M-TEER.

Our results demonstrated significant gradual shortening of fluoroscopy time over the last 10 years. This depicts a larger clinical experience with available devices, which is not only related to interventional techniques but also with the improvement of echocardiographic imaging techniques, which have evolved significantly over the last decade. In line with this, it may also be important to note that shorter procedural radiation is of great clinical importance not only for patients but also for all members of the team who are involved in M-TEER.

Short-term outcomes that include intra-hospital mortality, 30-day hospitalization, MACE, and mortality did not significantly change over the last 10 years even though there was a trend of reduction of all mentioned parameters. Very low incidence of mortality, hospitalization, and MACE after M-TEER is also very encouraging and underlines the safety of this method.

There are several important clinical implications of this study. It was observed that patients do not wait for the terminal phase of MR, characterized with largely dilated LV and LA, and undergo M-TEER earlier than before. This implies that M-TEER became the standard of care in patients with MR who are not eligible for operative treatment over the last decade. The trend clearly shows that the percentage of patients with primary MR and the percentage of patients with functional MR who were all treated with M-TEER are almost equal. The hospitalization time significantly reduced over the last 10 years, which is important for the cost-effectiveness of M-TEER. There is also a trend of the reduction of unfavorable shirt-term outcomes, which supports the high safety and significant benefits of this procedure.

### Limitations

There are some limitations to this study. Our findings were obtained from the local registry and not from a randomized clinical trial. Nevertheless, real-world data from the large population of patients who underwent M-TEER over the last decade provides the opportunity to observe trends and changes in this field for a long time. Patients with previous cardiac surgery were involved in the study, which might interfere with the final results. Nevertheless, this is a real population of patients with all common comorbidities. Therefore, the inclusion of all patients represents an important strength of this study.

## Conclusion

Our study included a large cohort of patients with MR who underwent M-TEER in our institution for 10 years. This revealed several important trends. The prevalence of patients with primary MR gradually increased over the last 10 years, whereas the prevalence of those with functional MR decreased. The burden of concomitant diseases remained virtually the same over this period. More recently, treated patients had lower surgical risk, better NYHA status, lower NT-pro-BNP, and a lower level of left heart remodeling than patients who were treated with this method at the beginning. This shows that patients nowadays are treated earlier before they develop all complications of MR-induced heart failure. The presented findings provided detailed insight into the epidemiological data of all MR patients undergoing M-TEER and revealed long-term trends for this unique interventional technique.

## Data Availability Statement

The raw data supporting the conclusions of this article will be made available by the authors, without undue reservation.

## Ethics Statement

The studies involving human participants were reviewed and approved by Ethical Committee of the Ulm University. The patients/participants provided their written informed consent to participate in this study.

## Author Contributions

MT and NN: conceptualization, methodology, and writing—original draft preparation. LS, TD, SM, and MK: investigation. WR: writing—review and editing. MT and WR: supervision. All authors contributed to the article and approved the submitted version.

## Conflict of Interest

The authors declare that the research was conducted in the absence of any commercial or financial relationships that could be construed as a potential conflict of interest.

## Publisher's Note

All claims expressed in this article are solely those of the authors and do not necessarily represent those of their affiliated organizations, or those of the publisher, the editors and the reviewers. Any product that may be evaluated in this article, or claim that may be made by its manufacturer, is not guaranteed or endorsed by the publisher.
